# Inducible over‐expression of cardiac Nos1ap causes short QT syndrome in transgenic mice

**DOI:** 10.1002/2211-5463.13520

**Published:** 2022-11-27

**Authors:** Monique Jänsch, Lubomir T. Lubomirov, Maximilian Trum, Tatjana Williams, Joachim Schmitt, Kai Schuh, Fatimunnisa Qadri, Lars S. Maier, Michael Bader, Oliver Ritter

**Affiliations:** ^1^ Department of Cardiology, Nephrology and Pneumology, Brandenburg Medical School University Hospital Brandenburg Germany; ^2^ Institute of Physiology Brandenburg Medical School Theodor Fontane Germany; ^3^ Department of Internal Medicine II University Hospital Regensburg Germany; ^4^ Comprehensive Heart Failure Center and Department of Internal Medicine I University Hospital Würzburg Germany; ^5^ Department of Pharmacology and Clinical Pharmacology Heinrich Heine University Düsseldorf Germany; ^6^ Institute of Physiology University of Würzburg Germany; ^7^ Max‐Delbrück‐Center for Molecular Medicine in the Helmholtz Association (MDC) Berlin Germany; ^8^ German Center for Cardiovascular Research (DZHK) Berlin Germany; ^9^ Charité University Medicine Berlin Germany; ^10^ Institute for Biology University of Lübeck Germany; ^11^ Department of Cardiology and Pneumology, Clinic for Internal Medicine I University Hospital Brandenburg Germany; ^12^ Faculty of Health Sciences, Joint Faculty of the Brandenburg University of Technology Cottbus – Senftenberg The Brandenburg Medical School Theodor Fontane and the University of Potsdam Germany

**Keywords:** APD90, L‐type calcium channel, NOS1AP, QT interval duration

## Abstract

Recent evidence demonstrated that alterations in the QT interval duration on the ECG are not only determined by mutations in genes for ion channels, but also by modulators of ion channels. Changes in the QT interval duration beyond certain thresholds are pathological and can lead to sudden cardiac death. We here focus on the ion channel modulator nitric oxide synthase 1 adaptor protein (Nos1ap). Whole‐cell patch‐clamp measurements of a conditional transgenic mouse model exhibiting cardiac‐specific Nos1ap over‐expression revealed a Nos1ap‐dependent increase of L‐type calcium channel nitrosylation, which led to increased susceptibility to ventricular tachycardias associated with a decrease in QT duration and shortening of APD_90_ duration. Survival was significantly reduced (60% after 12 weeks vs. 100% in controls). Examination of the structural features of the hearts of transgenic mice revealed constant heart dimensions and wall thickness without abnormal fibrosis content or BNP production after 3 months of Nos1ap over‐expression compared to controls. Nos1ap over‐expression did not alter cGMP production or ROS concentration. Our study showed that myocardial over‐expression of Nos1ap leads to the shortening of the QT interval and reduces the survival rate of transgenic animals, perhaps via the development of ventricular arrhythmias. We conclude that Nos1ap overexpression causes targeted subcellular localization of Nos1 to the CaV1.2 with a subsequent decrease of ADP_90_ and the QT interval. This causes detrimental cardiac arrhythmias in transgenic mice.

## Graphical abstract

We established and phenotyped a transgenic mouse model with cardiac‐specific over‐expression of nitric oxide synthase 1 adaptor protein (Nos1ap). We found increased nitrosylation of the L‐type calcium channel (CaV1.2) due to Nos1ap over‐expression, and patch‐clamp measurements showed a shortening of action potential duration. Nos1ap over‐expression resulted in a shortening of the QT interval on ECG, ventricular tachycardias and decreased survival rate without evidence of structural cardiac diseases.

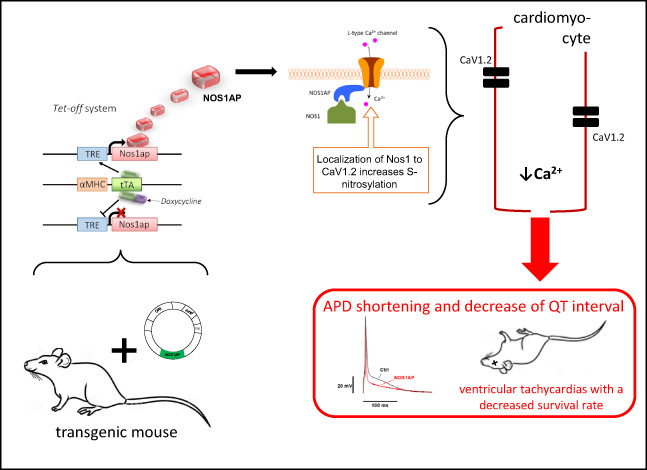

**Abbreviations**
APD_90_
: action potential duration at 90% of repolarization
BNP: brain natriuretic peptide
CaV1.2: voltage‐gated L‐type calcium channel‐ exclusively the cardiac subtype
I_Ca‐L_
: inward L‐type calcium current
LQTS: long QT syndrome
Nos1ap: nitric oxide synthase (Nos) 1 adaptor protein
PMCA4b: cardiac isoform 4b of plasma membrane calcium ATPase (PMCA)
QT interval: a defined part in an electrocardiogram (ECG) between the Q wave (start of depolarization) and the T wave (end of repolarization)
QTc interval: corrected QT interval according to the formula of Bazett
ROS: reactive oxygen species
SCD: sudden cardiac death
SD: standard deviation
SERCA2a: subtype 2a of SR calcium ATPase (SERCA) expressed in heart
SR: sarcoplasmic reticulum

Corrected QT interval variations predisposes individuals to ventricular tachycardia and sudden cardiac death (SCD). The long QT syndrome (LQTS) is a rare disorder of ventricular repolarization characterized by prolongation of the QTc interval on the electrocardiogram (ECG) [1, 2]. The QTc prolongation on the ECG is either an inherited syndrome or drug induced [3]. The inherited LQTS has a prevalence of approximately 1 in 2500 and is a major cause of SCD in the younger population [4, 5].

Since the landmark studies of Keating et al. [6] during the 1990s, the genetic origin of inherited LQTS has become clearer. Nevertheless, in 20% of families with LQTS diagnosis genetic screening is negative [7]. The likewise less common and less examined short QT syndrome (SQTS) is associated with genetic variants in genes encoding for potassium channel and as well for L‐type calcium channel (CaV1.2) [8]. Hence, it seems evident that the QTc interval has to be between certain thresholds to assure regular ventricular repolarization and avoid fatal clinical consequences.

The gating of different ion channels leads to ion currents across the sarcolemma. These currents constitute the membrane potential and cause the action potential (AP) and that influences the ECG [3]. We followed the hypothesis that not only mutations with monogenetic disease traits are causative for alterations in QT intervals and eventually SCD, but that modifiers of ion channel function can affect action potential duration and subsequently the QTc interval. Modification of ion channel function is complex in terms of molecular mechanisms behind it. GWAS have linked genetic polymorphisms in neuronal nitric oxide synthase 1 adaptor protein (NOS1AP) to variations in QTc interval duration [3, 9, 10, 11].

Our earlier investigations pointed out that Nos1 is considered to influence heart function due to cardioprotective effects and inhibition of cardiac contraction [12, 13]. We found higher Nos1 activity due to cardiac Nos1 over‐expression in mice, which resulted in higher NO production [12]. NO alters the activity of ion channels by post‐translational modification of cysteine residues. This alteration (called S‐nitrosylation) leads to reduced CaV1.2 open probability [14, 15].

CaV1.2, which is composed of the α1 subunit, β subunit and the α2δ subunit [16], mediates the inward L‐type calcium current (I_Ca‐L_) and contributes to the plateau phase of the cardiac action potential due to enhanced membrane depolarization. Inward I_Ca‐L_ also leads to calcium‐induced calcium release (CICR) in cardiac excitation‐contraction coupling.

We here investigated the relevance of the increased expression of Nos1ap acting as a CaV1.2 modulator via directing Nos1 to CaV1.2 (a simplified schematic drawing of interaction is depicted in Fig. 1A) on cardiac electrophysiology in a whole organism. Therefore, a conditional transgenic mouse with a heart‐specific Nos1ap over‐expression (Nos1ap^+^/αMHC‐tTA^+^) was generated. To the best of our knowledge, this is the first study *in vivo* confirming that over‐expression leads to the shortening of APD_90_ and QT interval.

**Fig. 1:**
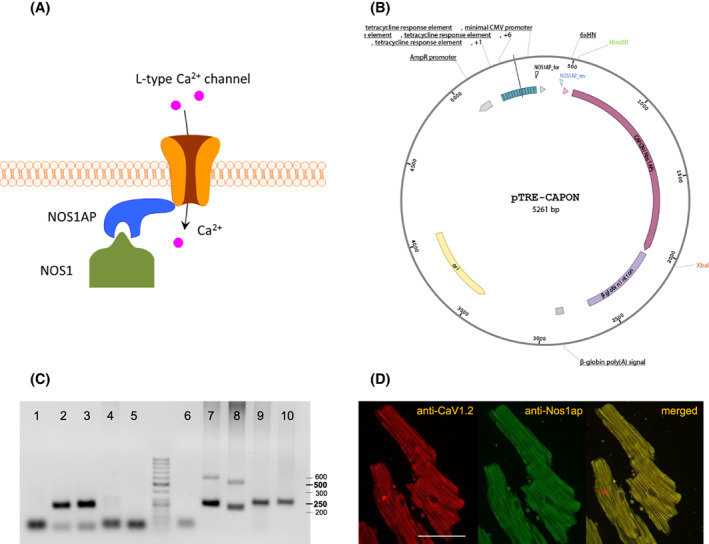
Nos1ap‐Nos1‐CaV1.2 interactions, main vector elements for establishment of transgenic founders, example for genotyping and proof‐of‐principle of inducing the Nos1ap over‐expression in murine cardiomyocytes. (A) Simplified schematic drawing of the Nos1‐Nos1ap‐CaV1.2 interactions. (B) Used pTRE‐6xHN vector with cDNA (1.509 kb) insert coding for murine Nos1ap with tetracycline responsive elements (TRE) and positions of used vector specific primers for genotyping. (C) Agarose gel of genotyping PCR with ladder. No template control in lanes 1/6, Nos1ap^+^ in lanes 2/3, Nos1ap^−^ in lanes 4/5), tetracycline‐controlled transactivator protein‐positive (tTA^+^) in lanes 7/8, tTA^−^ in lanes 9/10. (D) Confocal and immunofluorescence (with Nos1ap and CaV1.2 primary antibodies) imaging of ventricular cardiomyocytes from induced mice. Merged images show co‐localization of both proteins [bar = 50 μm].

## Materials and methods

### Animal studies

All experiments with mice conform with the German Animal Welfare Act and were approved by the Institutional Animal Care and Use Committees at Centre for Experimental Molecular Medicine (ZEMM) Wuerzburg (AZ: 55.2‐2531.01‐53/11) and Berlin State Office of Health and Social Affairs (G 0105/18). Transgenic founders were generated by the integration of pTRE‐6HN‐Nos1ap vector construct into the genome via microinjection to the pronuclei of fertilized FVB/N mouse oocytes [17]. The applied vector (Clontech, Takara Bio Inc., San Jose, CA, USA) was composed of a tetracycline response element (TRE) followed downstream by the gene of interest *Nos1ap* Isoform 1 (murine, Fig. 1B). These transgenic Nos1ap^+^ mice were mated with a second transgenic FVB.Cg‐Tg(Myh6‐tTA)6Smbf/J strain (The Jackson Laboratory, Bar Harbor, ME, USA) to obtain double transgenic Nos1ap^+^/αMHC‐tTA^+^ mice. Administration of doxycycline (100 mg·kg^−1^ food) leads to reversible binding to a tetracycline‐controlled transactivator protein (tTA) preventing activation of the TRE and coupled over‐expression of Nos1ap. For over‐expression, Nos1ap^+^/αMHC‐tTA^+^ mice of both sexes were induced by switching doxycycline chow to a standard diet without doxycycline 10 days before starting the experiments (*Tet‐off* system). Experimental start began at the age of 10–16 weeks and animals were housed in small groups of 4–6 animals at a temperature of 23 °C with a 12/12 h circadian rhythm and access to water/food *ad libitum*. The survival rate of induced and non‐induced (control) mice (*n* = 20) was determined during an observation time of 3 months and structural heart analyses were conducted afterwards.

### Genotyping

For genotyping DNA was extracted in accordance with the protocol of genomic DNA isolation kit, XIT for mouse tail (GBiosciences, St. Louis, MO, USA) and for PCR product amplification *Taq* DNA Polymerase (Qiagen) in combination with gene‐specific oligomer sets were used as follows: sense (Nos1ap) 5′‐TGAAAGTCGAGCTCGGTACC‐3′, antisense (Nos1ap) 5′‐CGTCAGCTGACTAGAGGATC‐3′, sense (tTA) 5′‐CGCTGTGGGGCATTTTACTTTAG‐3′, antisense (tTA) 5′‐CATGTCCAGATCGAAATCGTC‐3′, sense (tTA control) 5′‐CAAATGTTGCTTGTCTGGTG‐3′, antisense (tTA control) 5′‐GTCAGTCGAGTGCACAGTTT‐3′.

### Isolation of ventricular myocytes

Hearts of induced and non‐induced mice (both genders, age about 3 months) were freshly isolated using retrograde perfusion employing Langendorff technique. The cannulated heart was perfused for 2 min with a cold (4 ± 1 °C) tyrode solution followed by 6 min lysis solution (20 mL tyrode solution, 0.75 mg Liberase TM [Roche, Rotkreuz, Switzerland], 100 μL Trypsin [2.5%, Gibco, Thermo Fisher, Waltham, MA, USA], 25 μL of a 10 mm CaCl_2_). Thereafter mechanical cell digestion was prepared in a petri dish filled with stop solution (2.25 mL tyrode solution, 250 μL Bovine calf serum, 3.125 μL of 10 mm CaCl_2_) followed by a stepwise (8 min per step) increase (0.1, 0.2, 0.4, 0.8 mm) of calcium in CaCl_2_ solution with 5% bovine calf serum [18].

### Protein extraction, immunoblotting and co‐localization analyses

Isolated cells or tissue (10 mg) were homogenized for 15 min on ice in 600 μL lysis buffer by using a pellet micropestle (Eppendorf, Hamburg, Germany) followed by centrifugation at 12 000 ***g*** for 5 min. The supernatant contains the extracted proteins. Twenty microgram of proteins were denaturized in standard laemmli sample buffer for 5 min at 95 °C. Each sample was loaded on a lane of a 10% resolving and 4% stacking gel for SDS‐page (Mini‐PROTEAN Tetra electrophoresis system, Bio‐Rad). By using the Mini Trans‐Blot Cell system (Bio‐Rad Laboratories, Inc., Hercules, CA, USA) proteins were transferred to a methanol‐activated 0.45 μm PVDF (Amersham Hybon, Cytiva, Marlborough, MA, USA) membrane (constant 17 V, 14 h). The blotting buffer contains 25 boric acid and 2 EDTA (mm, pH 8.8) in water. PVDF was washed for 10 min in Tris‐Buffered Saline with 0.1% Tween (TBST) and 0.1% BSA (TBST/BSA) followed by blocking 1 h in 10% powdered milk diluted in TBST at room temperature (RT) and primary antibody incubation diluted in TBST with 3% BSA overnight at 4 °C, except for anti‐GAPDH (diluted in TBST with 5% powdered milk, incubation for 2 h at RT). Next, PVDF was washed twice in TBST/BSA, and subsequently, the antibodies were tagged by horseradish peroxidase‐conjugated secondary antibodies depending on host species diluted in TBST with 3% BSA, except tagging anti‐GAPDH (anti‐mouse was diluted in TBST with 5% powdered milk). Depending on protein amount for chemiluminescent detection ECL WB substrate or SuperSignal WestDura Substrate (both Thermo Scientific, Pierce, MO, USA) were utilized and pictures were conducted and quantified with the imager CHEMI Premium (VWR). The signal intensity was obtained after background correction. Co‐localization analyses were performed by using the immunoprecipitation starter pack consisting of protein A/G sepharose (Cytiva, Marlborough, MA, USA). In short; 70 μL protein A/G sepharose mix was incubated for 2 h with 3 μL monoclonal anti‐NOS1AP on a rotating mixer, and ~ 60 μg samples were added and gently mixed overnight at 4 °C. Negative controls contain beads and samples without antibodies. Pellet was washed twice with lysis buffer by centrifugation at 12 000 ***g*** for 30 s between each wash. The final pellet was mixed with laemmli sample buffer and heated for 4 min at 95 °C. The supernatant was loaded on SDS/page and transferred to a PVDF membrane. Immunoblotting was performed, respectively, with anti‐NOS1, anti‐CaV1.2, anti‐SERCA2a or anit‐PMCA4b antibody.

### Detection of protein S‐nitrosylation

The Pierce S‐Nitrosylation Western Blot Kit (Thermo Scientific) was used in accordance with the supplied manual. The kit starts with the blocking of free protein sulfhydryls followed by the reduction of bound S‐nitrosocysteines and labeling with a specific reagent for antibody detection. Immunoprecipitation using protein G sepharose (Cytiva) for isolation of labeled proteins was conducted, followed by a standard western blot with anti‐CaV1.2.

### Antibodies

#### Primary antibodies

ab190686, Abcam, Cambridge, UK, dilution 1 : 750 (WB), monoclonal, rabbit, anti‐NOS1AP MA1‐16757, Thermo Fisher, dilution 1 : 2000 (WB), monoclonal, mouse, anti‐GAPDH MA527717, Thermo Fisher, dilution 1 : 2000 (WB), monoclonal, mouse, anti‐CaV1.2 37‐2800, Thermo Fisher, dilution 1 : 1000 (WB), monoclonal, mouse, anti‐NOS1 MBS2090589, dilution 1 : 1000 (WB), monoclonal, mouse, anti‐NT Pro‐Brain Natriuretic Peptide sc‐20027, Santa Cruz Biotechnology, Dallas, TX, USA, dilution 1 : 300 (WB), monoclonal, mouse, anti‐PMCA4b sc‐376235, Santa Cruz, dilution 1 : 1000 (WB), monoclonal, mouse, anti‐SERCA2a.

#### Secondary antibodies

A27036, Thermo Fisher, dilution 1 : 2000 (WB), superclonal, goat/IgG, anti‐rabbit, HRP NXA931, Cytiva, dilution 1 : 2000 (WB), polyclonal, sheep/IgG, anti‐mouse, HRP.

### Electrocardiography with focus on QT interval

Electrocardiographic measurements were recorded for 1–5 min after light sedation of mice with isoflurane using needle electrodes. PowerLab 4/26 and Bio Amp modules (ADInstruments, Dunedin, New Zealand) were used for recordings in lead I configuration. ECG analysis was performed following automated algorithms with the labchart software (ADInstruments). The QT interval was calculated with the supplied ECG analysis tool, and invalid beats were excluded automatically. To monitor spontaneously occurring arrhythmias or cardiac events a mouse telemetry was conducted following a standard protocol. A transmitter ETA‐F10 (DSI, St. Paul, MN, USA) was implanted dorsal and after a postsurgical recovery phase of 7 days, measurements were conducted allowing free movements in special mouse telemetry cages. The cardiac potential changes were recorded for 24 h and sent to a receiver located on the bottom of the cages. The “holter”‐ ECGs were recorded and evaluated [19].

### Structural analysis of heart cross sections

Hearts of mice after 3 months of induction the cardiac Nos1ap over‐expression and respective controls were halved and heart tops were fixed in 4% formaldehyde overnight and dehydrated in ascending concentrations of ethanol (70–100%), incubated in paraffin using the tissue processor STP120 (Thermo Scientific) followed by paraffin‐embedding. Cooled paraffin wax blocks were sliced with a rotary microtome HM355S (Fisher Scientific) in 4 μm sections and mounted on slides. Before staining sections were deparaffinized and rehydrated as follows: three times in xylene, three times in 96% ethanol, 90% ethanol, 80% ethanol, 70% ethanol, 50% ethanol, 30% ethanol (each step for 5 min) and finally followed by another washing step for 10 min in water. For fibrosis, stained sections were incubated in 0.1% Picro‐Sirius‐Red solution (Morphisto, Frankfurt/Main, Germany) for 70 min in the dark followed by washing in 0.5% acetic acid solution. After staining all sections were dehydrated and coversliped using Eukitt Quick‐hardening mounting medium (Fa. Sigma Aldrich, St. Louis, MO, USA). Images were taken with the Aperio AT2 brightfield digital scanner (Leica Biosystems, Nussloch, Germany).

### Measurement of cyclic GMP levels, a second messenger of NO and determination of ROS concentration

Frozen hearts were homogenized for performing a cGMP immunoassay (R&D Systems, Wiesbaden, Germany) as described in Burkard et al. [12]. Followed by a standard ELISA assay according to the manufacturer's instructions. To assess the concentration of reactive oxygen species (ROS) in homogenized hearts of non‐induced or Nos1ap over‐expressing mice, the TAC‐Peroxyl assay kit (Applied Bioanalytical Labs) was used. The detection of ROS is based on the reaction of peroxyl radicals with an indicator molecule to generate chemiluminescense.

### Patch clamp experiments

Ruptured‐patch whole‐cell current clamp was used to measure action potentials in isolated murine cardiomyocytes on laminin‐coated recording chambers mounted on an inverted microscope (Zeiss Axio Observer) at room temperature. The pipette solution contained (mM) 122 K‐Aspartic Acid, 10 NaCl, 8 KCl, 1 MgCl_2_, 5 Mg‐ATP, 0.3 Li‐GTP and 10 HEPES (pH 7.2 with KOH). The bath solution contained (mM) 140 NaCl, 4 KCl, 1 MgCl_2_, 10 Glucose, 1 CaCl_2_ and 5 HEPES (pH 7.4 with NaOH). Fast capacitance was compensated in cell‐attached configuration, whereas membrane capacitance and series resistance were compensated after patch rupture. Access resistance was typically < 15 MOhm. Signals were filtered with 2.9 and 10 kHz Bessel filters and recorded with an EPC10 amplifier (HEKA Elektronik). APs were elicited using square current pulses of 1 nA and a duration of 1–4 ms at a frequency of 1 Hz. Only cells with excellent membrane potential stability and adequate overshoot upon each electrical stimulus were included in the analysis [20].

### Measurements of promoter activity of the human NOS1AP SNP rs16847548 (T/C) which had been associated with QTc prolongation

Neonatal rat cardiomyocytes were obtained from 1 to 3 days old Wistar rat pups and cell digestion was performed as described earlier in Burkard et al. (2005) [21]. Briefly, the ventricular tissue was digested in calcium and bicarbonate‐free Hank's medium with HEPES with 1.5 mg·mL^−1^ trypsin and 10 μg·mL^−1^ DNase. To inactivate trypsin, FCS was added to the resulting cell suspension, and cells were pelleted by centrifugation (5 min at 700 ***g***). The cells were resuspended in MEM/5 (minimum essential medium/5% fetal calf serum), and fibroblasts were removed by pre‐plating for 1 h and used for cell cultures. After pre‐plating, the supernatant (cardiomyocytes) was recovered and cells were plated in MEM/5 on six‐well plates at a density of 1 × 106 cells per well. Twenty‐four hours after plating, MEM/5 was replaced by MEM/1% FCS (MEM/1). Medium for cardiomyocytes contained 5‐Bromo‐2′‐deoxyuridine (0.1 mm) to suppress fibroblast growth. Fibroblast contamination of cardiomyocyte cultures was between 4–7% as regularly determined by immunohistochemical staining for troponin T. For luciferase assay, neonatal rat cardiomyocytes were transfected 48 h after preparation with the pGL3‐Promoter vector (Promega Corporation, Madison, WI, USA) using Lipofectamine LTX Reagent (Thermo Fisher). Each reaction mix containing 100 μL Opti‐MEM, 6 μL PLUS Reagent and 1 μg reporter plasmid (pDNA) and after 15 min incubation time another 100 μL Opti‐MEM and 4 μL Lipofectamine were added followed by another 15 min incubation. The pDNA contains the ~ 4 kb human NOS1AP fragment with the SNP rs16847548 upstream of the SV40 promoter in front of the luciferase gene and as control the wildtype NOS1AP. The pDNA‐lipid complex was added to cells in 6‐well plates for 4 h of transfection. Transfected cells were analyzed the next day after cell lysis and adding the Luciferase Assay Reagent (Promega) in accordance with the manufacturer's protocol. Luciferase activity was measured using a luminometer in relative light units (RLU) and six biological replicates were analyzed per each group.

### Solutions and buffers

Tyrode solution for retrograde perfusion (mm): 113 NaCl, 4.7 KCl, 0.6 KH_2_PO_4_, 0.6 Na_2_HPO_4_ × 2H_2_O, 1.2 MgSO_4_ × 7H_2_O, 0.032 Phenol red, 12 NaHCO_3_, 10 KHCO_3_, 10 HEPES, 30 Taurine, 10 BDM.

Lysis buffer for protein extraction (mm, pH 7.4): 300 NaCl, 100 NaH_2_PO_4_ × H_2_O, 10 Na_4_P_2_O_7_, 1 MgCl_2_ × 6 H_2_O, 50 Na_2_HPO_4_ and 10 EDTA, 1 PMSF, PhosSTOP phosphatase inhibitors (Roche) and cOmplete protease inhibitors (EDTA‐free, Roche).

### Statistics

Differences between experimental groups were evaluated using the *t*‐test in SPSS statistics (IBM). The paired respective unpaired *t*‐test was used depending on the type of examined variables. *P*‐values < 0.05 were considered in our experiments as a significant difference between the experimental groups. Values were given by mean and standard deviation (SD). The correlation coefficient was calculated by Pearson with the same SPSS software. The scatter plot graphs combined with bars for mean and SD were made with graphpad prism 9 (GraphPad Software, San Diego, CA, USA).

## Results

### Phenotyping of the transgenic mouse model with cardiac Nos1ap over‐expression

Double transgenic mice were genotyped with vector and tetracycline‐controlled transactivator protein (tTA) specific primers (Fig. 1C).

To confirm cardiac‐specific over‐expression in our *Tet‐off* mouse model immunofluorescence microscopy in cardiomyocytes (Fig. 1D) and protein expression analysis of different tissues were conducted 10 days after inducing the Nos1ap over‐expression.

**Fig. 2:**
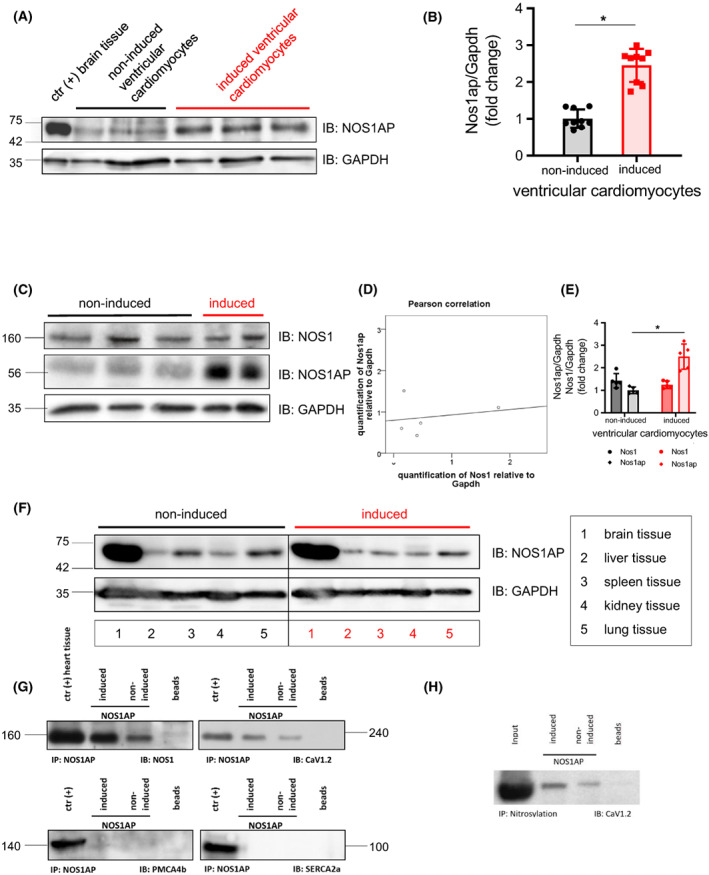
Nos1ap/Nos1 protein expression analysis of induced and non‐induced heart specific Nos1ap over‐expression in transgenic mice. Co‐localization of Nos1ap with Nos1, CaV1.2, SERCA2a and PMCA4b and nitrosylation of CaV1.2. (A) Example for heart specific protein expression of Nos1ap in different induced/non‐induced mice with statistic evaluation (B) normalized to Gapdh (*n* = 9, *P* < 0.05). (C) Western blots of Nos1ap/Nos1 for statistical correlation (D) by Pearson (coefficient = 0.207, not significant) normalized to Gapdh and (E) comparison of Nos1ap and Nos1 quantification in induced/non‐induced animals shows only significant (*n* = 5, *P* < 0.05) differential Nos1ap protein expression. (F) Nos1ap immunoblotting of different tissues in mice with cardiac Nos1ap over‐expression and controls, amount of loaded total protein was normalized using Gapdh as reference protein (*n* = 3). (G) Co‐immunoprecipitation of Nos1ap and immunoblotting with Nos1, CaV1.2, SERCA2a and PMCA4b (clockwise) by using heart tissue extracts from mice with or without Nos1ap over‐expression. (H) Nitrosylation assay of CaV1.2 in mice with and without Nos1ap over‐expression. Error bars indicate standard deviation (SD).

Western blot analysis confirmed induction as we found a 2.5‐fold higher Nos1ap expression in induced compared to non‐induced mice (Fig. 2A,B). Protein expression was normalized to Gapdh and values were given relative to Nos1ap expression in non‐induced animals. Our genetic modification resulted in higher expression of the full‐length isoform [15] of Nos1ap (~ 56 kDa). To analyze a putative connection between Nos1ap and Nos1 expression Pearson correlation analysis was calculated and we found no correlation (0.207) between Nos1ap and Nos1 expression levels in the myocardium (Fig. 2C–E). We confirmed the restriction of over‐expression to cardiac tissue by additional analysis of brain, liver, spleen, kidney and lung tissue in induced and non‐induced mice (*n* = 3). There was no significant increase in Nos1ap expression in tissues other than the heart in animals with Nos1ap over‐expression (Fig. 2F).

### Nos1ap co‐localization studies and protein S‐nitrosylation

To support our hypothesis that Nos1ap interacts with and modulates the L‐type calcium channel (CaV1.2) via directing Nos1 to the channel we performed protein–protein interaction studies. We found that Nos1ap specifically co‐immunoprecipitated with both Nos1 and CaV1.2 and we found no interaction with the other calcium channels SERCA2a and PMCA4b (Fig. 2G). We found higher S‐nitrosylation of CaV1.2 due to Nos1ap over‐expression (Fig. 2H).

### Arrhythmia and shortening of QT interval due to Nos1ap over‐expression

**Fig. 3:**
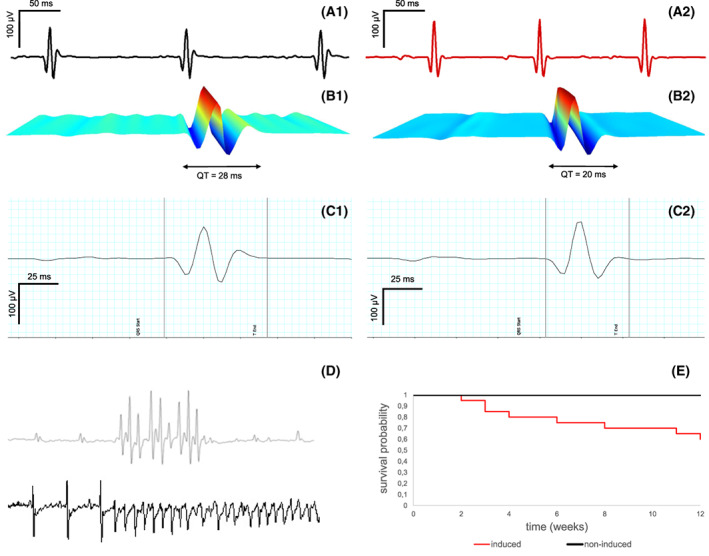
Electrocardiography in mice with/without cardiac Nos1ap over‐expression, Nos1ap over‐expression revealed significant decrease in QT interval, higher risk for arrhythmia with lower survival rates. (A1) Non‐induced and induced (A2) animals show sinus rhythm. (B1, C1) Non‐induced animals shows sinus rhythm, while Nos1ap over‐expressing mice (B2, C2) shows a significant (*n* = 6, *P* < 0.05) decrease in QT intervals as well as ventricular tachycardia or ventricular fibrillation (D). (E) Survival rate was reduced upon cardiac Nos1ap over‐expression monitored in a period of 12 weeks (*n* = 20) compared to control mice.

ECG of non‐induced and Nos1ap over‐expressing animals showed a basic sinus rhythm (Fig. 3A1,A2). We analyzed QT intervals in mice under sedation. There was a clear decrease in QT intervals (20 ± 1.9 vs. 28 ± 4.6 ms, *n* = 6, *P* < 0.05) in Nos1ap over‐expressing (Fig. 3B2,C2 and Table 1) compared to non‐induced animals (Fig. 3B1,C1). No significant changes were observed in the heart rate (481 ± 12 vs. 474 ± 11 beats per min, *n* = 6, *P* > 0.05) and in the QRS duration (16 ± 1 vs. 17 ± 1 ms, *n* = 6, *P* > 0.05) in Nos1ap over‐expressing compared to non‐induced animals (Table 1). As Roussel and co‐authors found there was only a weak relationship between RR and QT intervals in mice, therefore no correction formula (Bazett) was used [22].

**Table 1:** Analysis of QT interval duration and tachycardias. Surface ECG recordings were performed on six mice of each group (non‐induced and Nos1ap over‐expressing). From each mouse at least 10 heart beats (i.e. QT intervals) were calculated to a mean value. For assessment of rhythm disorders mice were under ECG telemetry. Non‐induced mice did not display any rhythm disorders. In contrast, Nos1ap over‐expressing mice showed a significantly increased number of arrhythmic events. Non‐sustained VT was defined as ventricular tachycardia < 10 s, sustained VT > 10 s. Ventricular fibrillation (VF) was defined as ongoing ventricular tachycardia without regular excitation. All mice with VF died thereof. bpm, beats per min

	Non‐induced	Nos1ap over‐expressing	*n*
QT duration	28 ± 4.6 ms	20 ± 1.9 ms*	6
Heart rate	474 ± 11 bpm	481 ± 12 bpm	6
QRS duration	17 ± 1 ms	16 ± 1 ms	6
Non sustained VT	–	3**	6 (non‐induced) 9 (induced)
Sustained VT	–	2**	6 (non‐induced) 9 (induced)
VF	–	4**	6 (non‐induced) 9 (induced)

Results: unpaired *t*‐test

* *P* < 0.05; surface ECG recordings in non‐induced vs. Nos1ap over‐expressing mice; unpaired *t*‐test

** *P* < 0.01; arrhythmias vs. no arrhythmias in non‐induced or Nos1ap over‐expressing mice; unpaired *t*‐test.

To determine *in vivo* electrophysiological features for a longer period of time, mouse telemetry was conducted for 24 h and cardiac events were recorded. Figure 3D and Table 1 displays non‐sustained ventricular tachycardia or ventricular fibrillation occurring in Nos1ap over‐expressing animals. No rhythm disorders were recorded in non‐induced mice (Table 1, *P* < 0.01 for arrhythmias vs. no arrhythmias in non‐induced or Nos1ap over‐expressing mice). The survival rate after 3 months of induction of Nos1ap over‐expression was significantly reduced (60% after 12 weeks vs. 100% in non‐induced mice) as demonstrated in Fig. 3E. There were no behavioral or nutritional abnormalities before the spontaneous death of the animal. Wildtype animals' behavior was unaltered due to withdrawal of the doxycycline from the diet for a lifetime, in consequence, the toxic effect of doxycycline can be excluded.

### Transgenic mice showed no structural heart disease and no differences in cGMP respective ROS concentration

**Fig. 4:**
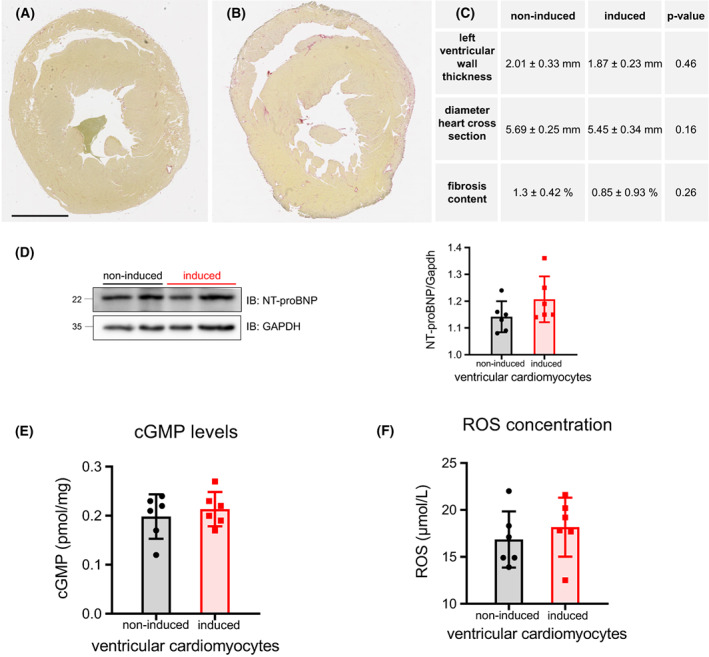
Phenotyping of murine hearts in induced and non‐induced animals including heart cross sections and level of fibrosis. Determination of NT‐proBNP/cGMP and ROS concentration. (A, B) Representative heart cross sections stained with Picro‐Sirius‐Red including averaged values (± SEM) for left ventricular wall thickness, diameter of heart cross section and collagen content as maker for fibrosis (C) of non‐induced (A, *n* = 8) and induced (B, *n* = 6) mice for Nos1ap over‐expression (*P* > 0.05) [bar = 2000 μm]. (D) Level of NT‐proBNP normalized to Gapdh (*n* = 6, *P* > 0.05). (E) Determination of cGMP levels and (F) ROS concentration in heart tissue of non‐induced and Nos1ap over‐expressing animals (*n* = 7, *P* > 0.05). Error bars indicate standard deviation (SD).

To exclude other cardiac diseases in Nos1ap over‐expressing mice reasonable for the reduced survival rate further studies were investigated. For evaluation of structural heart diseases due to cardiac specific Nos1ap over‐expression fibrosis staining of heart cross sections were conducted (Fig. 4A–C) using mice with 3 months of Nos1ap over‐expression compared to controls. Diameters of cross sections were not altered due to Nos1ap over‐expression (Fig. 4A–C, 5.45 ± 0.34 vs. 5.69 ± 0.25 mm in non‐induced). Histological examination revealed nearly constant wall thickness without abnormal fibrosis content (Fig. 4A–C) in both non‐induced (*n* = 8) and Nos1ap over‐expressing mice (*n* = 6). We conclude that there were no structural cardiac defects.

In addition, quantitative NT‐proBNP measurements gave no signs of heart failure due to induction of the transgenic phenotype (1.16 ± 0.8 in non‐induced to 1.25 ± 0.85 in Nos1ap over‐expressing mice, *P* > 0.05, Fig. 4D). We found no significant differences between cGMP production as we measured 0.199 ± 0.036 pmol·mg^−1^ in non‐induced and 0.204 ± 0.035 pmol·mg^−1^ in Nos1ap over‐expressing mice (*P* > 0.05), hence the cardiac NO amount was obviously not altered by Nos1ap over‐expression (Fig. 4E). The ROS concentration was not significantly changed in hearts of Nos1ap over‐expressing Nos1ap^+^/αMHC‐tTA^+^ mice compared with non‐induced animals (17.8 ± 3.7 vs. 17.2 ± 3.2 μmol·L^−1^, *P* > 0.05; Fig. 4F).

### APD_90_ changed upon Nos1ap expression in murine ventricular cardiomyocytes

As we found a clear decrease in QT intervals due to Nos1ap over‐expression and an interaction of Nos1ap with Nos1 and CaV1.2. We evaluated APD_90_ in isolated ventricular cardiac myocytes using whole‐cell patch‐clamp measurements. We postulated a modulation of CaV1.2 by increased S‐nitrosylation and finally a decreased cardiac action potential duration.

**Fig. 5:**
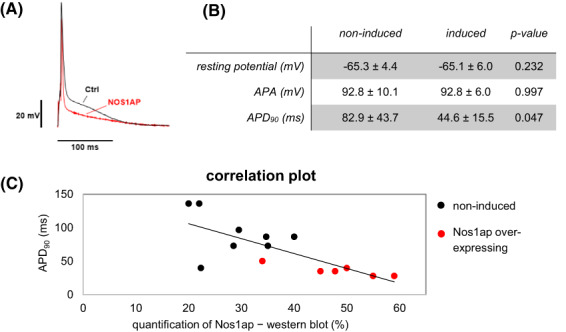
Analysis of action potential duration and Nos1ap expression in cardiomyocytes. (A) Example of traces of action potentials measured by patch‐clamp in ventricular myocytes with Nos1ap over‐expression (red) compared to controls. (B) Table of obtained patch‐clamp parameters (*n* = 6 of induced, *n* = 8 of non‐induced) and statistical evaluation using *t*‐test. APA, action potential amplitude. (C) Action potential duration at 90% of repolarization (APD_90_) in ventricular myocytes using whole‐cell patch‐clamp measurements and corresponding Nos1ap protein quantification normalized to Gapdh depicted in a scatter plot (*n* = 14).

Indeed, APD_90_ in induced transgenic Nos1ap over‐expressing mice was reduced compared to non‐induced littermates (Fig. 5A). Altered APD_90_ values due to enhanced Nos1ap expression did not lead to significant changes in resting membrane potential or action potential amplitude (Fig. 5B). Figure 5C give inverse correlation of cardiac Nos1ap protein expression in cardiac myocytes of different individuals and corresponding APD_90_ measurements. Action potential duration was decreased in hearts with higher Nos1ap protein expression (*r* ^2^ = 0.42).

### Minor allele of NOS1AP SNP rs16847548 (T/C) leading to decreased promoter activity in a luciferase assay

**Fig. 6:**
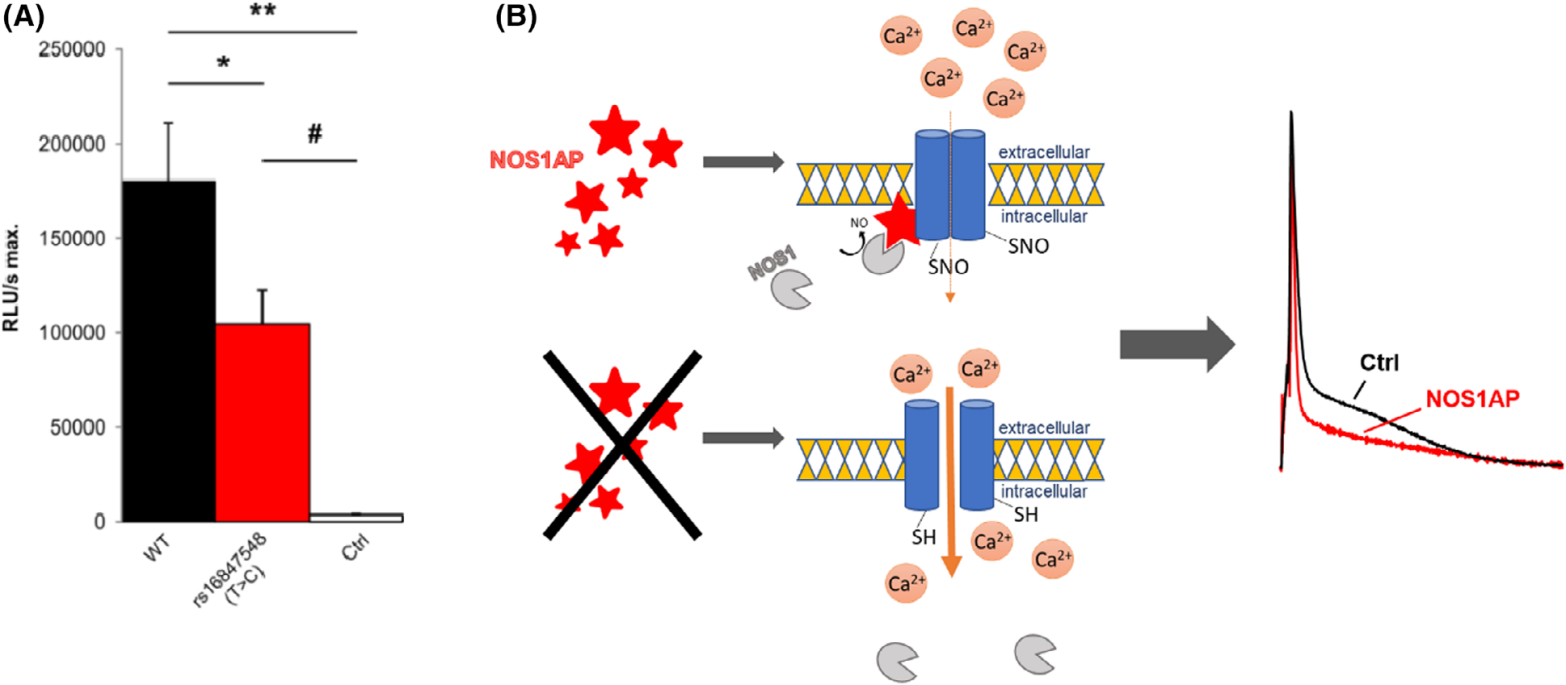
Investigations using a mutated promoter carrying the minor allele of SNP rs16847548 (T/C) in a viral Nos1ap luciferase assay and postulated mechanism of changed Nos1ap expression. (A) To investigate the effect of the NOS1AP single nucleotide polymorphism rs16847548 (T/C) on transcriptional activity we cloned the mutated promoter carrying rs16847548 (T/C) into a Luciferase reporter plasmid and compared transcriptional activity of the WT promoter and the mutated promoter in an unpaired *t*‐test. Un‐transfected cells served as controls. Transcriptional activity of the mutated NOS1AP promoter carrying rs16847548 indicated by Luciferase activity was significantly decreased compared to WT (*n* = 6 for each group, * for *P* < 0.05; # for *P* < 0.01; ** for *P* < 0.001. Error bars indicate standard deviation (SD). (B) Graphical abstract of postulated mechanism of changed Nos1ap expression in organism.

Since GWAS recently linked genetic variations in NOS1AP to QTc duration in otherwise healthy individuals and also in patients with known LQTS, additional investigations concerning the functional effect of the human SNP rs16847548 (T/C), which is located within the NOS1AP promoter region was conducted. Luciferase reporter assays demonstrated that this SNP significantly impaired transcriptional NOS1AP promoter activity resulting in less transcriptional activity in rat cardiomyocytes (Fig. 6A). In conclusion, the rs16847548 SNP may result in less NOS1AP expression in affected individuals. This attenuates NOS1AP mediated inhibition of CaV1.2 and leads subsequently to QT prolongation effects (Fig. 6B).

## Discussion

The main findings of this work are as follows: (a) in a transgenic mouse model with conditional Nos1ap over‐expression there was a significant shortening of the QT interval in the ECG. (b) Nos1ap over‐expressing mice had a strong susceptibility to ventricular arrhythmias and decreased survival rates. (c) Nos1ap interacted with and was co‐localized to Nos1 and CaV1.2. (d) Nos1ap over‐expression caused S‐nitrosylation of CaV1.2 and a decrease in APD_90_.

### Transgenic mice with cardiac conditional Nos1ap over‐expression

Genetic variations with the potential for modifying arrhythmia susceptibility may occur within the coding or noncoding regions of a gene. Further, genetic variability occurs in many forms from single nucleotide and copy number variants to larger chromosomal aberrations. But alterations of ion channel function could also be achieved via posttranslational modification.

Among other LQTS‐susceptibility genes that code mostly for ion channels, NOS1AP was shown in clinical studies to exhibit multiple common nucleotide variants that are highly associated with QTc interval duration even in the general population [10, 11, 23, 24]. A focused search in LQTS families ultimately yielded multiple NOS1AP variants that are highly predictive of the severity of the disease phenotype [11]. Remarkably, not only did these variants predict pro‐arrhythmic risk in familial LQTS, but they were also found to correlate with drug‐induced forms of the disorder [25, 26].

NOS1AP is supposed to be a spacer protein, which tethers NOS1 to CaV1.2 in cardiac myocytes. However recent experimental approaches using over‐expression and knockdown strategies to modulate the myocardial levels of NOS1AP resulted in divergent findings. One study reported that adenoviral‐mediated over‐expression of NOS1AP in isolated ventricular myocytes of guinea pigs shortened action potential duration (APD) by inhibiting I_Ca‐L_ [15]. In contrast, a study in zebrafish models documented APD shortening in response to NOS1AP knockdown [27]. Sugiyama and colleagues established a transgenic mouse model with a Nos1ap deletion leading to prolongation of the QT interval after oxidative stress but without ECG alterations under baseline conditions [28]. Our hypothesis pursues the assumption that cardiac Nos1ap over‐expression leads to a decrease of the QT interval. Our results provided evidence that Nos1ap in our rodent model of transgenic Nos1ap over‐expression guides Nos1 to the CaV1.2. Nos1 enzymatic function was not altered by Nos1ap as cGMP levels were similar in both induced and non‐induced littermates. Nevertheless, there was a significant increase in CaV1.2 S‐nitrosylation in Nos1ap over‐expressing mice, indicating that local accumulation of Nos1 (caused by targeted guiding via Nos1ap) at the CaV1.2 led to an increase in S‐nitrosylation. This is in line with recent findings from Treuer et al. [29] who investigated the impact of Nos1ap silencing in neonatal cardiomyocytes. They also confirmed the co‐localization of Nos1ap with CaV1.2 and found reduced S‐nitrosylation due to Nos1ap silencing using siRNA. Likewise, they proposed a decrease in S‐nitrosylation of CaV1.2 as a trigger for cardiac arrhythmias [28].

Our results give no evidence for structural heart diseases in this model [30, 31]. LV diameter, NT‐proBNP as a surrogate marker for heart failure and extent of myocardial fibrosis were similar in Nos1ap over‐expressing mice and controls (Fig. 3). As already mentioned above we could not detect a transcriptional effect of Nos1ap over‐expression on Nos1 protein levels. Similar results were reported by Sugiyma in a Nos1ap‐KO model [27]. In contrast, Ronchi et al. found a correlation in NOS1AP/NOS1 protein expression in that lower NOS1AP levels conditioned lower NOS1 levels. However, this was assessed in cardiac myocytes derived from human pluripotent stem cells from patients harboring KCNQ1‐A341V mutations causing LQTs with concomitant NOS1AP SNPs as minor and major variants (rs16847548 and rs4657139) [32].

Similar with previous observations, the interaction of Nos1ap with Nos1 and CaV1.2 seems very clear [15, 29], whereas its impact in cardiac function is not completely understood. The principle of Nos1ap mediated guiding of Nos1 to targets was already described earlier in neurons. Nos1ap (earlier named Capon) was seen in neurons as an adaptor protein that targets NOS1 to a small G‐protein called RASD1 to activate signal transduction pathways. S‐nitrosylation and inhibition of the NMDA receptor were enhanced due to PSD‐95 acting as a bridge by binding NOS1 and co‐localization to the channel. These mechanisms are involved in learning and memory processes [33]. SNPs in the promoter region of NOS1AP for instance are correlated to schizophrenia [34]. Accordingly, it appears that modulation of ion channel function by targeted guiding of modulators and adaptor proteins is a common feature in cellular electrophysiology. Beigi et al. confirmed the adaptor function of Nos1ap in the heart by directing Nos1 to CaV1.2 in the sarcolemma after myocardial infarction (MI) to enhance nitrosylation of the channel. The accompanied reduction of intracellular calcium levels after MI is considered as protection against cardiac injury [35]. So Nos1ap seems to influence subcellular compartmentation and in that way modulates CaV1.2 comparable with PSD‐95 in the brain.

### Electrocardiographic analysis of Nos1ap over‐expressing phenotype and related electrical disorder in cardiomyocytes and analysis of minor NOS1AP allele

As noted before NOS1AP is suggested to transport and tether NOS1 to CaV1.2 leading to alteration of cardiac electrophysiology [15]. We here found a decrease of APD_90_ paralleled by a decrease of the QT interval and spontaneous ventricular tachycardias with a decreased survival rate (60% after 3 months) in the Nos1ap over‐expressing mice. As mice were monitored with a score sheet daily without any phenotypical changes before death, a sudden cardiac death was suspected here.

As I_Ca‐L_ is important for depolarization during the plateau phase of the AP, we confirmed the interaction of Nos1ap with Nos1 and CaV1.2 and observed a short QT interval due to cardiac Nos1ap over‐expression. Consequently, Nos1ap seems to increase the effect of Nos1. Similarly, Ronchi et al. [32] have presented that NOS1 inhibition leads to APD_90_ prolongation independent of repolarizing outward currents, but due to decreased S‐nitrosylation respectively inhibition of CaV1.2.

These results follow an older study from our group with over‐expression of Nos1. We hypothesized at that time that the close proximity of Nos1 and certain effector molecules like CaV1.2 has an impact on myocardial repolarization. Accordingly, in adult isolated cardiac myocytes, CaV1.2 density was significantly decreased in the Nos1 over‐expressing cells [12, 13]. In these previous experiments with conditional Nos1 over‐expression, we found co‐localization of Nos1 with Serca2a and Pmca4b and also accumulation in mitochondria with subsequent functional alterations such as a decrease in myocardial contractility. In the present animal model of transgenic Nos1ap over‐expression, however, there was selective guiding of Nos1 to CaV1.2 via Nos1ap.

As subcellular NOS1 targeting occurs via specific binding sites and shuttling mechanisms it seems clear that Nos1ap over‐expression only addresses CaV1.2 as a specific target. In the case of PMCA4b, syntrophin was previously described as a linker protein to tether NOS1 to PMCA4b [36]. Similarly, the heat shock protein 90 (HSP90) was described as a carrier for NOS1 to the outer mitochondrial membrane [13]. With regard to SERCA2a, there were no previous reports on potential NOS1AP binding. In Co‐IP experiments, we were not able to detect an interaction.

Previous reports correlated production of reactive oxygen species (ROS) by NADPH oxidase or by xanthine oxidoreductase (XOR) to NOS1 derived NO [13, 28, 37]. We could not detect alterations in ROS levels depending on Nos1ap expression levels as Nos1ap expression had no impact on myocardial Nos1 levels in general and NADPH oxidase and XOR were obviously no subcellular targets for NOS1AP.

As NOS1AP expression levels obviously affected NOS1 localization, we investigated the transcriptional effect of the minor allele of the SNP rs16847548 which is located in the NOS1AP promoter and was associated with LQTS [9]. Using luciferase assays this SNP revealed a decreased transcriptional activity of the NOS1AP promoter providing an explanation for elongation of QTc intervals in that less NOS1AP causes less NOS1‐mediated inhibition of I_CaL_.

To what extent does subcellular NOS1 targeting alter fundamental myocyte properties such as calcium influx? Presumably, subcellular localization and translocation during different disease states is of major importance for the functional effects of NOS1. In our (older) Nos1 over‐expressing model we found interaction of NOS1 with SR calcium ATPase and additionally with CaV1.2 with subsequent biological effects [12, 13]. In our Nos1ap over‐expressing model we observed alterations only with the CaV1.2 function. We interpret these findings in that specific subcellular targeting of effector molecules is crucial for precise work, especially during cardiac excitation which is very sensitive to disorders. We believe that NOS1AP is biologically rather inactive but serves as a targeting molecule for confined intracellular localization, in this case, NOS1 to CaV1.2. If intracellular localization is disturbed by decreased or increased expression (by transgenic approaches in animals or genetics in humans) this might result in fatal rhythm disorders.

In an attempt to understand the electrocardiographic alterations, i.e. the shortening of the QT interval, we assessed the action potential duration in isolated adult cardiac myocytes. In our Nos1ap over‐expressing mice we found a decrease in APD_90_. We then tried to investigate calcium and potassium currents using patch clamp approaches. Unfortunately, we were not able to record either calcium or potassium currents in a significant number. The overall yield of viable isolated adult cardiac myocytes from our transgenic strains was low. Additionally, the viability of the single myocytes was also poor, so serial measurements in single cells did not work properly. That was surprising since a previous model from our group with identical genetic background (ref. 12 in the manuscript) but Nos1 and not Nos1ap over‐expression was not hampered by similar problems. Nonetheless, immunohistochemical stainings, nitrosylation levels of the CaV1.2 and ADP_90_ recordings were confirmative for our hypothesis of Nos1/Nos1ap/CaV1.2 interaction.

## Conclusion

Given that the over‐expression of Nos1ap *per se* has a detrimental effect on CaV1.2 conductance, our study suggests that targeting of the protein expression of Nos1ap could be a relevant future strategy for prevention of cardiac arrhythmia.

This may further explain, at least in part, negative results in genetic testing in the evaluation of inherited arrhythmia syndromes.

## Conflict of interest

The authors declare no conflict of interest.

## Author contributions

MJ and OR conceived the study and designed experiments; MJ, LTL and OR wrote the manuscript; TW and OR established the transgenic mouse model; MJ and FQ performed the histology; MJ, LTL and TW performed western blots and Co‐IPs; JS and KS performed electrocardiography; MT, LSM and MJ performed and analyzed the whole‐cell patch‐clamp measurements; Animal facility and biometrical planning was conducted by MB, MJ and OR. All authors are responsible for the contents and have read and approved the manuscript.

## Data Availability

The data that supports the findings of this study are available in Figs 1, 2, 3, 4, 5, 6 and Table 1.
